# Population Heterogeneity in Iron Biomarkers by Age, Sex, Menopausal Status, and Race in Healthy U.S. Adults: A Cross-Sectional Analysis from the All of Us Research Program

**DOI:** 10.3390/nu18101522

**Published:** 2026-05-10

**Authors:** Rola S. Zeidan, Jae Jeong Yang, Ruina He, Erta Cenko, Alicia M. Mohr, Anna Picca, Stephen D. Anton, Stefano Cacciatore

**Affiliations:** 1 Department of Physiology and Aging, College of Medicine, University of Florida, Gainesville, FL 32611, USA; santon@ufl.edu; 2 Department of Health Outcomes and Biomedical Informatics, College of Medicine, University of Florida, Gainesville, FL 32611, USA; 3 Department of Surgery, College of Medicine, University of Florida, Gainesville, FL 32611, USA; yang.jae@surgery.ufl.edu (J.J.Y.); alicia.mohr@surgery.ufl.edu (A.M.M.); 4 Cancer Institute, University of Florida Health, Gainesville, FL 32611, USA; 5 Department of Biostatistics, University of Florida, Gainesville, FL 32611, USA; ruinahe@ufl.edu; 6 Department of Epidemiology, College of Public Health and Health Professions, University of Florida, Gainesville, FL 32611, USA; ertacenko@phhp.ufl.edu; 7 Department of Medicine and Surgery, LUM University, 70010 Casamassima, Italy; picca@lum.it; 8 Fondazione Policlinico Universitario “Agostino Gemelli” IRCCS, 00168 Rome, Italy

**Keywords:** iron biomarkers, ferritin, transferrin saturation, reference intervals, population heterogeneity, demographic differences, menopause, race, nutritional assessment, All of Us Research Program

## Abstract

**Background/Objectives**: Blood iron biomarkers are commonly interpreted using fixed clinical reference intervals, although iron metabolism varies by age, sex, menopausal status, and race. This study aimed to characterize the distribution of iron biomarkers across demographic subgroups and to examine their distribution relative to commonly used reference intervals. **Methods**: In this cross-sectional study, 7990 adults (≥18 years) from the All of Us Research Program were classified as premenopausal women, postmenopausal women, men < 53 years, or men > 56 years. Serum iron, ferritin, total iron-binding capacity, unsaturated iron-binding capacity, and transferrin saturation (TSAT) were summarized across groups and compared descriptively with commonly used reference intervals. **Results**: Iron biomarkers varied across demographic groups. Mean serum iron was 82.3 µg/dL overall, with lower levels in premenopausal women (79.5 ± 36.7 µg/dL) and higher levels in men < 53 years (86.8 ± 41.8 µg/dL). Mean TSAT was 25.6% and generally located toward the lower end of commonly used reference ranges. Ferritin showed substantial variability, with higher mean levels in younger men (258.0 ± 581.0 ng/mL) and lower levels in premenopausal women (102.1 ± 224.9 ng/mL). Premenopausal women had higher iron-binding capacity, whereas older men had lower values. Black participants had lower serum iron and TSAT but higher ferritin compared with White participants. A substantial proportion of participants had values outside commonly used reference intervals, particularly for serum iron and TSAT. **Conclusions**: Iron biomarker distributions differ across demographic subgroups and may not be fully reflected by commonly used reference intervals. These findings highlight the importance of context-specific interpretation and underscore the need for further studies to evaluate the applicability of current reference intervals across diverse populations.

## 1. Introduction

Iron is a critical element for oxygen transport, energy production, and multiple metabolic processes [1,2]. Because of its redox activity, iron homeostasis is tightly regulated, as both deficiency and overload may have detrimental health consequences, including anemia, oxidative stress, and organ dysfunction [1,2,3]. Circulating blood biomarkers such as serum iron (indicating circulating iron availability), ferritin (corresponding to body iron storage), total iron-binding capacity (TIBC) and unsaturated iron-binding capacity (UIBC) (reflecting iron-binding capacity), and transferrin saturation (TSAT) (representing the proportion of transferrin saturated with iron), are routinely integrated in clinical practice to evaluate iron deficiency and iron overload [2]. Interpretation of these measures relies on laboratory reference intervals intended to support the distinction between typical and potentially clinically relevant values [4].

However, iron biomarker levels vary systematically according to age, sex, reproductive status, and race/ethnicity [1,2,5,6], and are influenced by hormonal regulation, inflammation, and dietary intake [2,7]. Premenopausal women generally exhibit lower iron stores than postmenopausal women and men, largely due to menstrual blood loss, whereas iron stores tend to increase after menopause and with aging [8,9,10]. Similarly, population-level differences in iron biomarkers have been consistently observed across racial and ethnic groups [11,12,13,14,15]. These sources of variability may complicate the interpretation of iron biomarkers when uniform reference intervals are applied across heterogeneous populations.

Despite these well-recognized sources of variation, clinical interpretation of iron biomarkers commonly relies on fixed reference intervals, which vary across laboratories and are often derived from relatively small and homogeneous samples [16,17,18,19,20]. Emerging evidence has questioned whether current reference intervals adequately reflect variation across diverse populations, and recent reviews have highlighted both methodological heterogeneity in their derivation and limited representation of underrepresented groups in the underlying data [17,19,21]. A clearer understanding of the distribution of iron biomarkers across demographic subgroups in contemporary populations is therefore needed to support more informed interpretation of these measures.

The All of Us Research Program provides a large and demographically diverse U.S. cohort to examine population distributions of iron biomarkers and address gaps in their descriptive epidemiology [22]. In this study, we aimed to (1) characterize differences in the distribution of serum iron, ferritin, TIBC, UIBC, and TSAT across age, sex, menopausal status, and race/ethnicity; (2) evaluate whether these differences persist after adjustment for sociodemographic and health-related factors; and (3) describe the distribution of biomarker values in relation to commonly used clinical reference intervals.

## 2. Materials and Methods

### 2.1. Study Design and Population

This cross-sectional study used version 8 Controlled Tier data from the All of Us Research Program, a large, nationwide research initiative supported by the National Institutes of Health that aims to enroll a diverse population across the United States to advance precision medicine. The program integrates electronic health records, survey data, and biospecimens to support research on a wide range of health outcomes [22]. All participants provided written informed consent at enrollment. The study was approved by the Institutional Review Board of the All of Us Research Program (protocol v9, 20 March 2020) [23] and was conducted in accordance with the principles of the Declaration of Helsinki.

For the present analyses, we included participants aged ≥ 18 years with at least one available iron biomarker measurement. Participants with anemia were identified using standardized condition concepts within the All of Us data model, mapped to standard terminologies (e.g., SNOMED) from source vocabularies such as ICD-9-CM and ICD-10-CM, corresponding to clinically relevant diagnostic categories (Table A1). Similarly, participants with gastrointestinal inflammatory diseases (including Crohn’s disease, ulcerative colitis, diverticulitis, and gastroenteritis), pregnancy or lactation, malignancies, hemochromatosis, or other genetic iron-overload disorders were identified using standardized condition concepts and excluded. Individuals reporting use of iron, vitamin B12, or folate supplements during the study period were also excluded. To enhance comparability across participants, laboratory measurements obtained outside a one-year window around study enrollment were excluded. This time window was selected to minimize temporal misclassification between biomarker measurements and baseline covariates, particularly with respect to age-based group definitions. Menopausal status was determined using electronic health record (EHR)-derived clinical information within the All of Us Research Program, based on standardized condition concepts mapped from source vocabularies (including ICD-9 and ICD-10) into a common data model using standardized terminologies indicative of natural or surgical menopause (Table A2). In addition, self-reported menopausal status was captured through participant responses to the survey question “Why did your periods stop?”. Women were classified as postmenopausal if menopause-related clinical records were present and/or if survey responses indicated cessation of menses due to natural or surgical menopause prior to the time of biomarker measurement.

**Figure 1:**
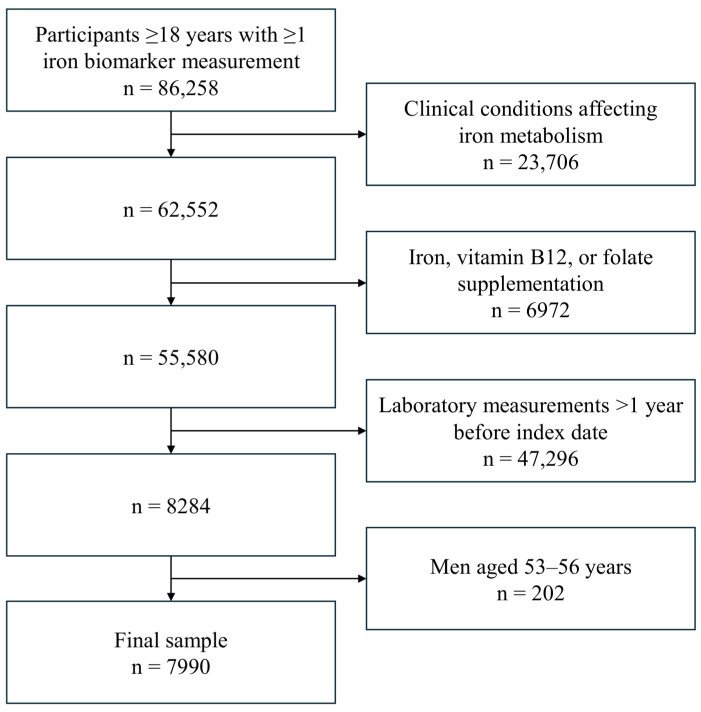
Flowchart of sample selection. Clinical conditions affecting iron metabolism include anemia, inflammatory diseases, pregnancy/lactation, malignancies, and iron-overload disorders.

To minimize misclassification, women with evidence of menopausal transition during the study period and those with discordant information across data sources were excluded. In addition, men aged 53–56 years were excluded to create clearly separated age categories based on a predefined cut-off of 55 years, avoiding overlap between groups and reducing potential misclassification around boundary values [24,25]. After applying these criteria, the final analytic cohort included 7990 participants (Figure 1). Participants were classified into four prespecified groups: premenopausal women, postmenopausal women, men younger than 53 years, and men older than 56 years. Analyses of iron biomarkers by race were restricted to White and Black or African American participants due to heterogeneity and small sample sizes in other racial categories.

### 2.2. Iron Biomarkers and Reference Intervals

We analyzed serum iron (µg/dL), ferritin (ng/mL), total iron-binding capacity (TIBC, µg/dL), unsaturated iron-binding capacity (UIBC, µg/dL), and transferrin saturation (TSAT, %), as obtained from the All of Us Research Program dataset. Reference intervals were not used for classification but are provided in Table A3 as descriptive benchmarks for contextual interpretation, based on commonly reported ranges in U.S. clinical laboratories, including those from the National Board of Medical Examiners (https://www.nbme.org/) accessed on 6 April 2026.

### 2.3. Statistical Analysis

Participant characteristics were summarized using descriptive statistics. Continuous variables are presented as mean ± standard deviation, and categorical variables as counts and percentages. Differences between groups were assessed using analysis of variance (ANOVA) for continuous variables and the chi-squared test for categorical variables. Biomarker values were evaluated in relation to their corresponding reference intervals, and the proportions of values below, within, or above these intervals were calculated for descriptive purposes. Associations between study group and each iron biomarker were examined using linear regression models, with men aged > 56 years as the reference group. Models were adjusted for race, ethnicity, highest educational attainment, annual household income, health insurance status, alcohol use, smoking status, and self-reported general health. Regression coefficients (β) with 95% confidence intervals (CIs) were reported. All analyses were conducted using R software (version 4.5.0; R Foundation for Statistical Computing, Vienna, Austria). All statistical tests were two-sided, and *p*-values < 0.05 were considered statistically significant.

## 3. Results

### 3.1. General Characteristics of the Sample

Demographic characteristics differed across the four study groups, including race/ethnicity, educational attainment, income, insurance status, smoking, alcohol use, and self-rated health (all *p* < 0.001). Overall, the study population was predominantly White, with a high level of educational attainment and health insurance coverage. Women were more represented than men, particularly in the younger group. Complete demographic data are provided in Table 1.

### 3.2. Iron Biomarker Differences by Age and Sex

**Figure 2:**
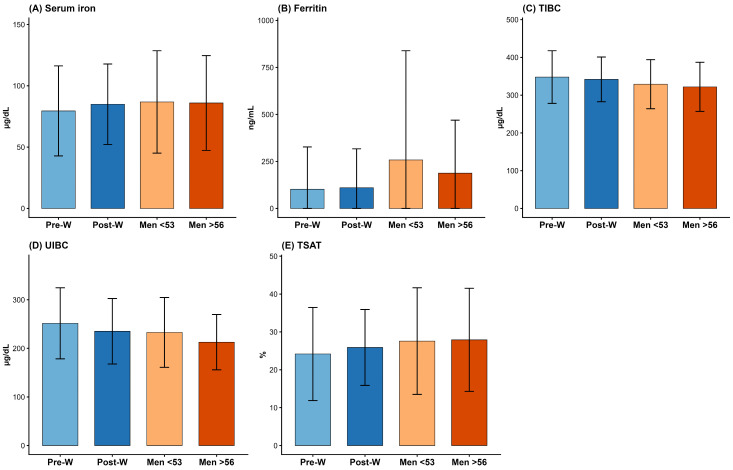
Sex- and age-related differences in circulating iron biomarkers. Bar graphs show mean ± standard deviation values for (**A**) serum iron, (**B**) ferritin, (**C**) total iron-binding capacity, (**D**) unsaturated iron-binding capacity, and (**E**) transferrin saturation across four groups: premenopausal women, postmenopausal women, men < 53 years, and men > 56 years. Abbreviations: Pre-W, premenopausal women; Post-W, postmenopausal women; TIBC, total iron-binding capacity; TSAT, transferrin saturation; UIBC, unsaturated iron-binding capacity.

Mean levels of iron biomarkers across study groups are illustrated in Figure 2. Serum iron levels were slightly lower in premenopausal women (79.5 ± 36.7 µg/dL) compared with postmenopausal women (85.0 ± 32.9 µg/dL) and men, whose values were similar across age groups (86.8 ± 41.8 µg/dL in men < 53 years and 86.0 ± 38.7 µg/dL in men > 56 years). Ferritin levels showed more pronounced differences across groups, with substantially lower values in women (102.3 ± 224.9 ng/mL in premenopausal and 110.1 ± 207.0 ng/mL in postmenopausal women) compared with men, particularly younger men (258.0 ± 581.0 ng/mL), while older men had intermediate values (187.7 ± 281.6 ng/mL). TIBC and UIBC were consistently higher in premenopausal women (347.9 ± 69.8 µg/dL and 251.5 ± 73.1 µg/dL, respectively) and decreased across postmenopausal women and men, with the lowest values observed in older men. TSAT was lower in women (24.2 ± 12.3% in premenopausal and 25.9 ± 10.0% in postmenopausal women) and higher in men, with similar values in younger and older groups (27.6 ± 14.1% and 27.9 ± 13.6%, respectively).

**Figure 3:**
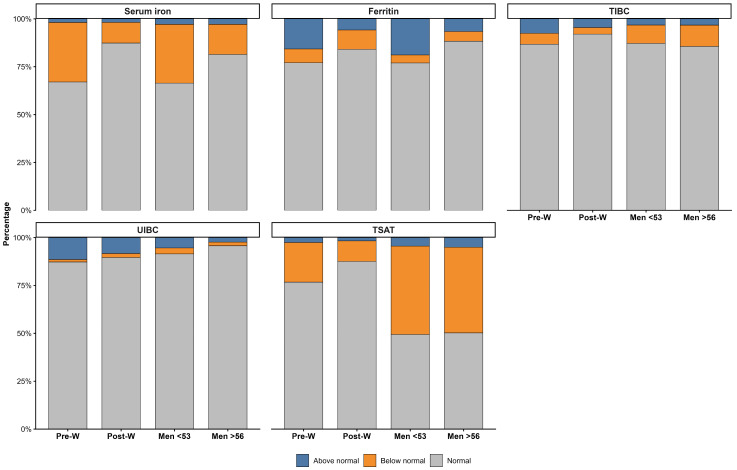
Distribution of iron biomarker categories across age and sex groups. Stacked bar plots show the proportion of values above normal (blue), below normal (orange), and within the reference interval (grey) for serum iron, ferritin, total iron-binding capacity, unsaturated iron-binding capacity, and transferrin saturation across four groups: premenopausal women, postmenopausal women, men < 53 years, and men > 56 years. Abbreviations: Pre-W, premenopausal women; Post-W, postmenopausal women; TIBC, total iron-binding capacity; TSAT, transferrin saturation; UIBC, unsaturated iron-binding capacity.

The distribution of biomarker categories across groups is shown in Figure 3. For serum iron, values below the reference interval were observed in 31.1% of premenopausal women and 30.7% of men < 53 years, compared with 10.8% of postmenopausal women and 15.7% of men > 56 years. Values above the reference interval were infrequent across all groups (≤2.8%). For ferritin, values above the reference interval were present in 15.7% of premenopausal women and 18.9% of men < 53 years, compared with 5.8% of postmenopausal women and 6.6% of men > 56 years. Values below the reference interval ranged from 4.2% in men < 53 years to 10.1% in postmenopausal women. For TIBC, values above the reference interval were observed in 7.5% of premenopausal women, 4.5% of postmenopausal women, 3.2% of men < 53 years, and 3.3% of men > 56 years. Values below the reference interval were reported in 5.7%, 3.4%, 9.5%, and 11.1% of these groups, respectively. For UIBC, most values were within the reference interval across all groups (>87%). Values above the reference interval were observed in 11.5% of premenopausal women, 8.3% of postmenopausal women, 5.4% of men < 53 years, and 2.4% of men > 56 years. For TSAT, values below the reference interval were observed in 20.7% of premenopausal women, 10.7% of postmenopausal women, 46.1% of men < 53 years, and 44.8% of men > 56 years. Values above the reference interval were present in ≤5.0% across groups.

### 3.3. Iron Biomarker Differences by Race

**Figure 4:**
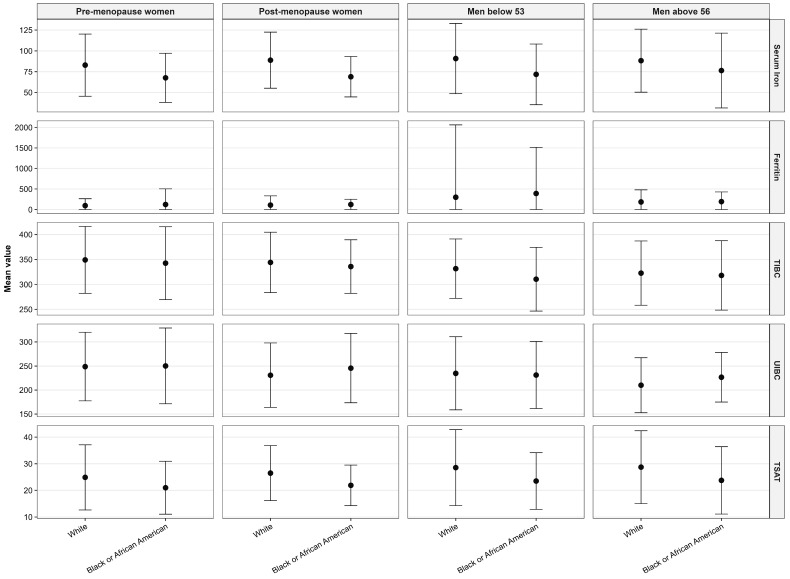
Iron biomarkers stratified by race, sex, and age group. Mean values with standard deviations are shown for serum iron, ferritin, total iron-binding capacity, unsaturated iron-binding capacity, and transferrin saturation across racial groups (White and Black or African American) and stratified by sex and age categories (pre-menopause women, post-menopause women, men aged < 53 years, and men aged > 56 years). Abbreviations: TIBC, total iron-binding capacity; TSAT, transferrin saturation; UIBC, unsaturated iron-binding capacity.

Differences in iron biomarkers were observed across racial groups (Figure 4). Compared with White participants, Black individuals had lower serum iron levels across all subgroups, with values of 67.6 vs. 82.9 µg/dL in pre-menopausal women, 68.9 vs. 88.8 µg/dL in post-menopausal women, 71.8 vs. 90.7 µg/dL in men aged < 53 years, and 76.3 vs. 88.2 µg/dL in men aged > 56 years (all *p* < 0.001). A similar pattern was observed for transferrin saturation (TSAT), which was lower in Black participants across all subgroups (e.g., 21.0% vs. 24.9% in pre-menopausal women; *p* < 0.001). Ferritin concentrations were higher in Black participants compared with White participants, with substantial dispersion across groups. TIBC values were generally lower in Black participants across most subgroups (e.g., 342.6 vs. 349.1 µg/dL in pre-menopausal women and 310.5 vs. 331.6 µg/dL in men aged < 53 years), while UIBC values were broadly similar between groups, with modest differences across subgroups.

### 3.4. Associations with Demographic and Clinical Characteristics

In multivariable linear regression models adjusting for demographic and clinical factors (Table 2), associations between study groups and iron biomarkers were observed. Compared with men aged > 56 years (reference group), pre-menopausal women had lower serum iron (β −5.66 µg/dL, 95% CI −8.15 to −3.17; *p* < 0.001), whereas no significant differences were observed in post-menopausal women or younger men. For ferritin, pre-menopausal and post-menopausal women showed lower levels compared with the reference group (β −87.38 ng/mL, 95% CI −103.98 to −70.78; *p* < 0.001 and β −76.96 ng/mL, 95% CI −102.99 to −50.93; *p* = 0.005, respectively), while younger men had higher ferritin levels (β 47.96 ng/mL, 95% CI 24.26 to 71.66; *p* < 0.001). TIBC was higher in women, particularly in pre-menopausal individuals (25.25 µg/dL, 95% CI 20.27 to 30.23; *p* < 0.001), with smaller and borderline differences in younger men. A similar pattern was observed for UIBC, which was significantly higher in pre-menopausal women (β 38.84 µg/dL, 95% CI 27.65 to 50.03; *p* < 0.001) and in younger men (19.31 µg/dL, 95% CI 3.49 to 35.13; *p* = 0.017). Finally, TSAT was lower in both pre-menopausal and post-menopausal women (β −3.61%, 95% CI −4.47 to −2.75; *p* < 0.001 and −2.19%, 95% CI −3.60 to −0.78; *p* = 0.002, respectively), while no significant differences were observed in younger men.

## 4. Discussion

In this large, contemporary U.S. cohort, iron biomarkers showed substantial heterogeneity across age, sex, menopausal status, and race/ethnicity that was not fully reflected by existing clinical reference intervals. Although mean biomarker values generally fell within conventional ranges, a considerable proportion of participants had values outside commonly used reference intervals, particularly for serum iron and transferrin saturation. These findings highlight incomplete differences between population-level distributions and current reference standards.

Patterns observed across demographic groups were consistent with known biological and life-course differences in iron metabolism as follows. Premenopausal women exhibited lower ferritin, serum iron, and TSAT and higher iron-binding capacity compared with men, whereas postmenopausal women showed intermediate profiles. Among men, ferritin and UIBC differed between younger and older groups, while other biomarkers were relatively stable. These differences persisted after adjustment for sociodemographic and health-related factors, supporting the presence of systematic variation across demographic subgroups. Our findings are consistent with prior studies showing higher ferritin and lower TIBC in postmenopausal women and older men, reflecting changes in iron balance across the life course [9,10,26,27].

Historically, many reference intervals have been derived using statistical approaches based on the central distribution of selected populations, such as mean ± 2 standard deviations or percentile-based cutoffs [28,29]. As a result, commonly used ranges, including ferritin levels of 15–80 ng/mL and TSAT values of approximately 20–50%, have been incorporated into clinical practice [19,21,30]. However, these intervals were often established in relatively small or homogeneous samples and without systematic consideration of key demographic factors such as age, sex, or race/ethnicity, or of relevant biological modifiers such as inflammation [17]. More recent studies have proposed refined intervals based on larger datasets [31,32,33], yet substantial variability remains in how reference values are defined and applied. In this context, fixed cutoffs may not fully reflect the diversity of iron biomarker distributions observed across contemporary populations. Taken together, these observations highlight a persisting gap in the evidence base underlying reference interval definition and suggest the need for further research to better characterize iron biomarker variability across diverse groups.

Beyond age- and sex-related differences, distinct patterns emerged across racial and ethnic groups, although analyses were limited to White and Black or African American participants due to heterogeneity and small sample sizes in other groups. Black participants had lower serum iron and TSAT but higher ferritin compared with White participants. These findings are consistent with previous analyses, including those from the National Health and Nutrition Examination Survey, although the present data do not allow mechanistic interpretation [11,12,34]. A similar pattern has been described in prior studies showing that African American populations may exhibit higher ferritin levels despite a higher prevalence of iron deficiency [35]. Potential explanations have been proposed in the literature, including chronic low-grade inflammation, comorbidity burden, and differences in healthcare access, although these factors could not be directly assessed in the present study [36]. Accordingly, the observed patterns should be interpreted descriptively. In this context, race and ethnicity should be interpreted as proxies for a range of exposures, including dietary patterns, access to care, comorbidity burden, and inflammatory status, rather than as markers of inherent biological differences [35,37]. Differences in dietary habits across sex and racial groups may also contribute to the observed variability in iron biomarkers. Prior studies have shown that men tend to consume higher amounts of red and processed meat, whereas women more frequently report higher intake of plant-based foods and dietary fiber, which may influence iron bioavailability [38,39]. Similarly, racial differences in dietary quality, micronutrient intake, and food access have been documented, potentially affecting both iron intake and absorption [40]. These factors, together with known differences in eating behaviors and food environments, may partly underlie the heterogeneity observed in circulating iron indices and further emphasize the importance of contextual interpretation of iron biomarkers.

From a clinical and nutritional perspective, these findings underscore the need for cautious interpretation of iron biomarkers across diverse populations. Early identification of altered iron status remains clinically relevant, although its interpretation should be contextualized within individual and population characteristics [41]. Similar biomarker values may reflect different underlying contexts depending on demographic characteristics, and reliance on uniform thresholds may not fully capture this variability. However, the present study is descriptive, and the clinical significance of values outside conventional reference intervals cannot be determined without longitudinal data or outcome-based validation. Future studies linking biomarker distributions to incident anemia, functional outcomes, and disease-specific endpoints will be essential to clarify their clinical relevance.

Within this broader clinical context, these findings suggest the importance of context-aware interpretation of blood iron biomarkers, taking into account age, sex, reproductive status, and race/ethnicity. This perspective is consistent with recent calls to re-examine how clinical decision limits are applied across diverse populations [42,43,44]. Ferritin results warrant particular caution. As both a marker of iron stores and an acute-phase reactant, ferritin may be influenced by inflammation, liver disease, and metabolic factors, especially in older adults. In the absence of systematically available inflammatory markers, we were unable to distinguish elevated ferritin related to iron status from that associated with other processes [27,45]. Accordingly, ferritin should be interpreted within its broader biological and clinical context, and its variability across populations represents an important area for further investigation.

Several limitations should be considered when interpreting these findings. First, the cross-sectional design precludes causal inference and does not allow assessment of within-person changes over time. Second, variability in laboratory assays across collection sites may have introduced measurement heterogeneity. Third, inflammatory markers such as C-reactive protein were not uniformly available, limiting the interpretation of ferritin levels. Given that ferritin is influenced by inflammation, liver function, and metabolic status, its values should be interpreted with caution, particularly in the absence of systematically measured inflammatory markers. Fourth, menopausal status was derived from a combination of EHR-based clinical information and self-reported survey data. Although this approach likely improved classification accuracy compared with the use of a single data source, residual misclassification may still have attenuated differences between premenopausal and postmenopausal groups. Fifth, although exclusion criteria were applied to reduce the influence of conditions known to affect iron metabolism, residual confounding cannot be excluded, as unmeasured or subclinical factors may still have influenced biomarker levels. Sixth, although the cohort is diverse, certain groups may be over- or underrepresented, potentially affecting generalizability. Seventh, iron biomarkers were assessed at a single time point, which may not fully capture intra-individual variability over time. Finally, the absence of longitudinal outcome data limits our ability to assess the clinical relevance of biomarker distributions or to determine whether values outside reference intervals are associated with meaningful health outcomes.

Despite these limitations, the strengths of this study lie in the large, demographically diverse cohort and the integration of laboratory and sociodemographic data, which enabled detailed characterization of iron biomarker distributions across multiple population subgroups. By leveraging real-world data, this work provides insight into variability beyond conventional reference thresholds and suggests the importance of evaluating reference intervals in contemporary, demographically representative cohorts. Greater harmonization of laboratory practices, together with consideration of demographic and physiological factors, may contribute to improved understanding of iron biomarker variability across populations [24,46,47].

## 5. Conclusions

Iron biomarker distributions vary substantially across demographic subgroups and are not fully captured by commonly used reference intervals. These findings highlight the importance of considering demographic and clinical context when interpreting iron biomarkers and suggest the need for further research to evaluate their clinical relevance and inform future refinement of reference standards.

## Figures and Tables

**Table 1:** Baseline characteristics of the study population by age and sex groups, presented as counts and percentage (%).

Characteristics	Total Sample *n* = 7990	Women *n* = 5356		Men *n* = 2634	
		Pre-Menopause *n* = 4654	Post-Menopause *n* = 702	Men < 53 Years *n* = 926	Men > 56 Years *n* = 1708
Race					
Black or African American	1198 (15%)	751 (16.1%)	90 (12.8%)	135 (14.6%)	222 (13%)
White	5809 (72.7%)	3285 (70.6%)	563 (80.2%)	605 (65.3%)	1356 (79.4%)
Others	983 (12.3%)	618 (13.3%)	49 (7%)	186 (20.1%)	130 (7.6%)
Ethnicity					
Hispanic or Latino	315 (3.9%)	211 (4.5%)	12 (1.7%)	61 (6.6%)	31 (1.8%)
Not Hispanic or Latino	7675 (96.1%)	4443 (95.5%)	690 (98.3%)	865 (93.4%)	1677 (98.2%)
Highest educational attainment					
Less than high school	246 (3.1%)	126 (2.7%)	21 (3%)	36 (3.9%)	63 (3.7%)
High school or GED	1085 (13.6%)	588 (12.6%)	87 (12.4%)	176 (19%)	234 (13.7%)
Some college	2286 (28.6%)	1423 (30.6%)	197 (28.1%)	240 (25.9%)	426 (24.9%)
College degree	2182 (27.3%)	1296 (27.8%)	181 (25.8%)	252 (27.2%)	453 (26.5%)
Advanced degree	2077 (26%)	1164 (25%)	199 (28.3%)	207 (22.4%)	507 (29.7%)
Annual household income					
<25,000$	1412 (17.7%)	879 (18.9%)	108 (15.4%)	186 (20.1%)	239 (14%)
25,000–50,000$	1267 (15.9%)	743 (16%)	120 (17.1%)	128 (13.8%)	276 (16.2%)
50,000–75,000$	1010 (12.6%)	619 (13.3%)	83 (11.8%)	103 (11.1%)	205 (12%)
75,000–100,000$	846 (10.6%)	500 (10.7%)	81 (11.5%)	94 (10.2%)	171 (10%)
100,000–150,000$	1101 (13.8%)	642 (13.8%)	92 (13.1%)	124 (13.4%)	243 (14.2%)
>150,000$	1178 (14.7%)	642 (13.8%)	107 (15.2%)	164 (17.7%)	265 (15.5%)
Not reported/not available	1177 (14.7%)	630 (13.5%)	111 (15.8%)	127 (13.7%)	309 (18.1%)
Health insurance status					
Yes	7684 (96.2%)	4492 (96.5%)	689 (98.1%)	872 (94.2%)	1631 (95.5%)
No	195 (2.4%)	95 (2%)	6 (0.9%)	38 (4.1%)	56 (3.3%)
Not reported/not available	112 (1.4%)	68 (1.5%)	7 (1.0%)	16 (1.7%)	21 (1.2%)
Alcohol use					
Any	5683 (71.1%)	3367 (72.3%)	517 (73.6%)	652 (70.4%)	1147 (67.2%)
None	1506 (18.8%)	782 (16.8%)	131 (18.7%)	163 (17.6%)	430 (25.2%)
Not reported/not available	802 (10.0%) *	506 (10.9%)	54 (7.7%)	111 (12.0%)	131 (7.7%) *
Smoking status					
Current smoker	3114 (39%)	1652 (35.5%)	263 (37.5%)	354 (38.2%)	845 (49.5%)
Non smoker	4649 (58.2%)	2894 (62.2%)	406 (57.8%)	551 (59.5%)	798 (46.7%)
Former smoker	227 (2.8%)	108 (2.3%)	33 (4.7%)	21 (2.3%)	65 (3.8%)
Self-reported general health					
Excellent	544 (6.8%)	296 (6.4%)	50 (7.1%)	56 (6%)	142 (8.3%)
Very good	2159 (27%)	1243 (26.7%)	233 (33.2%)	211 (22.8%)	472 (27.6%)
Good	2796 (35%)	1621 (34.8%)	264 (37.6%)	320 (34.6%)	591 (34.6%)
Fair	1740 (21.8%)	1039 (22.3%)	120 (17.1%)	223 (24.1%)	358 (21%)
Poor	509 (6.4%)	306 (6.6%)	20 (2.8%)	84 (9.1%)	99 (5.8%)

* Percentages may not sum to exactly 100.0% because of rounding. Alcohol use was categorized as a binary variable and did not capture quantity or frequency of consumption, which may limit the interpretation of its association with iron biomarkers. All comparisons across groups were statistically significant (*p* < 0.001).

**Table 2:** Unadjusted and adjusted linear regression models for iron biomarkers across study groups.

Outcome	Term	Unadjusted β (95% CI)	*p*-Value	Adjusted β (95% CI) *	*p*-Value
Serum iron	Pre-menopause women	−6.47 (−8.98 to −3.96)	<0.001	−5.66 (−8.15 to −3.17)	<0.001
	Post-menopause women	−1.04 (−5.37 to 3.29)	0.637	−1.39 (−5.62 to 2.84)	0.521
	Men < 53 years	0.83 (−2.80 to 4.46)	0.653	2.28 (−1.31 to 5.87)	0.213
	Men > 56 years	Reference	—	Reference	—
Ferritin	Pre-menopause women	−85.44 (−101.83 to −69.05)	<0.001	−87.38 (−103.98 to −70.78)	<0.001
	Post-menopause women	−77.58 (−103.69 to −51.47)	0.006	−76.96 (−102.99 to −50.93)	0.005
	Men < 53 years	55.89 (32.39 to 79.39)	<0.001	47.96 (24.26 to 71.66)	<0.001
	Men > 56 years	Reference	—	Reference	—
TIBC	Pre-menopause women	25.89 (21.01 to 30.77)	<0.001	25.25 (20.27 to 30.23)	<0.001
	Post-menopause women	19.79 (11.60 to 27.98)	<0.001	18.68 (10.47 to 26.89)	<0.001
	Men < 53 years	7.01 (−0.10 to 14.12)	0.054	7.03 (−0.22 to 14.28)	0.057
	Men > 56 years	Reference	—	Reference	—
UIBC	Pre-menopause women	38.84 (27.96 to 49.72)	<0.001	38.84 (27.65 to 50.03)	<0.001
	Post-menopause women	22.50 (0.67 to 44.33)	0.044	20.28 (−1.89 to 42.45)	0.073
	Men < 53 years	20.09 (4.82 to 35.36)	0.010	19.31 (3.49 to 35.13)	0.017
	Men > 56 years	Reference	—	Reference	—
TSAT	Pre-menopause women	−3.72 (−4.58 to −2.86)	<0.001	−3.61 (−4.47 to −2.75)	<0.001
	Post-menopause women	−2.01 (−3.44 to −0.58)	0.006	−2.19 (−3.60 to −0.78)	0.002
	Men < 53 years	−0.32 (−1.57 to 0.93)	0.612	−0.07 (−1.32 to 1.18)	0.915
	Men > 56 years	Reference	—	Reference	—

* Adjusted for sex, race, ethnicity, household income, insurance status, educational attainment, self-reported health, alcohol consumption, and smoking status. Abbreviations: TIBC, total iron-binding capacity; TSAT, transferrin saturation; UIBC, unsaturated iron-binding capacity.

## Data Availability

Data are available through the All of Us Research Program Researcher Workbench, subject to registration, training, and project approval. Access to individual-level data is restricted due to privacy and ethical considerations. Researchers may request access at https://www.researchallofus.org/.

## Appendix A

**Table A1:** Clinical categories of anemia based on ICD-9-CM and ICD-10-CM definitions, mapped to standardized condition concepts in the All of Us data model.

ICD-9-CM Code	Description	ICD-10-CM Code	Description
280.0–280.9	Iron deficiency anemia	D50.0–D50.9	Iron deficiency anemia (dietary, chronic blood loss, unspecified)
285.1	Acute posthemorrhagic anemia	D62	Acute anemia due to recent blood loss
285.2	Anemia of chronic disease	D63.0–D63.8	Anemia in chronic disease (including neoplastic disease, chronic kidney disease and other chronic diseases)
285.9	Anemia, unspecified	D64.0–D64.9	Other and unspecified anemia
281.0–281.9	Other nutritional anemias	D51–D53	Vitamin B12, folate, and other nutritional anemias
282.0–282.9, 283	Hemolytic anemias	D55–D59	Hereditary and acquired hemolytic anemias
284.0–284.9	Aplastic anemia	D61	Aplastic and other bone marrow failure syndromes
238.7	Neoplasm of uncertain behavior of other lymphatic and hematopoietic tissues	D46	Myelodysplastic syndromes

**Table A2:** Clinical categories of menopausal status based on ICD-9-CM and ICD-10-CM definitions, mapped to standardized condition concepts in the All of Us data model.

ICD-9-CM Code	Description	ICD-10-CM Code	Description
627.2	Symptomatic menopausal or female climacteric states	N95.1	Menopausal and female climacteric states
627.3	Postmenopausal atrophic vaginitis	N95.2	Postmenopausal atrophic vaginitis
627.8	Other specified menopausal disorders	N95.8	Other specified menopausal and perimenopausal disorders
627.9	Unspecified menopausal disorder	N95.9	Unspecified menopausal and perimenopausal disorder
		Z78.0	Asymptomatic menopausal state

**Table A3:** Iron biomarkers commonly used U.S. clinical laboratories’ reference intervals across age and sex according to the National Board of Medical Examiners.

		Men		Women	
Biomarker	Unit	<55 Years	>55 Years	Pre-Menopause	Post-Menopause
Serum iron	µg/dL	65–176	50–170	60–170	50–170
Ferritin	ng/mL	20–300	20–500	12–150	20–300
TSAT	%	20–50	20–50	15–50	15–50
TIBC	µg/dL	250–450	250–450	250–450	240–450
UIBC	µg/dL	111–343	111–343	111–343	111–343

Abbreviations: TIBC, total iron-binding capacity; TSAT, transferrin saturation; UIBC, unsaturated iron-binding capacity.
